# Management of undiagnosed pheochromocytoma with acute appendicitis

**DOI:** 10.1186/1749-7922-4-35

**Published:** 2009-10-15

**Authors:** Mustapha Bensghir, Abderhmane Elwali, Salim Jaafar Lalaoui, Noureddine Drissi kamili, Hassan Alaoui, Jawad Laoutid, Hicham Azendour, Hicham Balkhi, Charqui Haimeur, Mohamed Atmani

**Affiliations:** 1 Service of anaesthesiology, Military Hospital Mohammed V Rabat, Morocco; 2 Intensive Care Unit and Emergency, Military Hospital Mohammed V Rabat, Morocco

## Abstract

The authors reported and discussed management a case of undiagnosed pheochromocytoma suspected because the catastrophic hemodynamic changes in a patient with an acute appendicitis.

## 

Letter to editor:

Pheochromocytoma is a rare catecholamine-secreting tumor. A proportion of patients are diagnosed at the time of incidental surgery, when induction of anaesthesia may precipitate an hypertensive crisis. In this situation, mortality is close to 80% [1]. The authors report a case of an undiagnosed pheochromocytoma patient with an acute appendicitis.

A 17 years old man was scheduled for acute appendicitis. The patient's cardiovascular examination was normal, arterial blood pressure was 135/65 mmHg and heart rate was 85 beats/min. A crush-induction (Propofol 3 m/kg, célocurine 1 mg/kg) was used. Anaesthesia was maintained with sevoflurane in a mixture of nitrous oxide and oxygen. Five minutes after resection the appendicitis, just as washing the abdominal cavity, the blood arterial pressure abruptly increased up to 210/110 mmHg and heart rate increased to 200 beats/min. Anaesthesia was deepened. Medication errors were ruled. There was no skeletal muscle rigidity and the body temperature was 37°c. EtCO2 and airway pressure had not changed and kaliemia was 4.5 mmol/L. An arterial catheter was placed to be able to rapidly detect and treat any hypertension crisis. The arterial pressure continued to rise to 220/120. Heart rate varied from 100 to 140 beats/min. The diagnosis of pheochromocytoma was suspected. The anaesthesiologist and surgeon decided to interrupt the surgery. IV incremental dose of nicardipine and esmolol were given and resulted in arterial pressure of 125/50 mmHg and heart rate of 70 beats/min. Once the patient stabilized, closing the fascia and the skin was effected. Infusion of nicardipine was started and adjusted according to the blood pressure. In intensive care unit, aggressive therapy included nicardipine, propanolol and hydration was continued. After extubation the pression was stabilized by nicardipine 6 mg per hour and propanolol 40 mg twice per day. Abdominal injected computerized tomography showed a unilateral suprarenal mass. Measure in 24 h urine collection disclosed metanephrine of 0,57 mg/24 H (0,04-0,3 mg/24 H) confirming the diagnosis of pheochromocytoma. The patient was discharged home on day 5, with nicardipine 20 mg twice daily, and propanolol 40 mg only once a day. Two months later, the patient had a resection of suprarenal tumour. Pathology examination confirmed the diagnosis of pheochromocytoma. Control blood pressure was normal; any treatment was administered.

Few reports of intraoperative presentation of pheochromocytoma are reported in the literature [2-5]. In this case the excessive secretion of catecholamines can have devasting consequences because any preparation has realised. A significant complication is being directly related to preoperative increase in systolic blood pressure [6]. Noxious stimuli, such as venous catheterization, tracheal intubation, skin incision, anaesthetics drugs and palpation of the tumour or abdominal exploration will start the hypertension crisis by releasing catecholamine of the tumours. In our case the differential diagnosis considered included pheochromocytoma and carcinoid syndrome. Malignant hyperthermia, thyrotoxic crisis were believed to be less likely in this clinical picture. Succinylcholine may cause mechanical stimulation of the tumour by fasciculation's. In our case probably washing the abdomen by surgeon, not succinylcholine administration has start the crisis because it occurred a long time after induction.

The reported sensitivity and specificity for metanephrines/chatecolamines in the 24 hr urines and are respectively 97% and 69%, and 86% and 88%. CT scan sensitivity is 88%. Magnetic resonance or 131I-MIBG scintigraphy showed a sensitivity of 100%. Plasma levels of free metanephrines have sensitivity or 99% and specificity of 89% [7]. In our case, the diagnosis has been made by elevated urinary metanephrines and the localization has identified by CT. Pathology examination of the tumor confirmed the diagnosis of pheochromocytoma. In our hospital the dosage of free plasma metanephrines it's not available and the access to the Magnetic resonance or 131I-MIBG scintigraphy remains limited.

The intra-operative incidental presentation of the pheochromocytoma represents usually a dramatic event, being a therapeutic challenge with a very difficult control of the intra-operative blood pressure and often carrying a tragic outcome. The hypertensive crisis should be immediately controlled. A α and β-adrenergic blockers should be considered. It is essential that hypertension is controlled with a rapidly acting α-adrenergic blocker before instituting any β-adrenergic receptor blockade. Suppression of B-adrenoceptor-mediated cardiac sympathetic in the absence of adequate arteriolar dilatation may precipitate acute pulmonary oedema [8]. Different drugs have been successfully used [2,5,9] table 1. In our case the use of the nicardipine, esmolol and intravascular hydratation volume have rapidly and effectively controlled the crisis. In a case of undiagnosed pheochromocytoma with acute appendicitis reported by Tarent [2], the surgery has cancelled and medicals treatment was administered. The medical treatment of acute appendicitis has no clear. In our case the surgery was almost finished and there remained only washing and closing.

**Table 1:** Intra-operative controlled hypertension and drugs

**Authors**	**Drugs**
Holldack HJ [5]	Nitroglycerine
	Esmolol
	Clonidine
James MFN [9]	Magnesium sulfate
Tarant NS [2]	Nitroprusside
	Phentolamine
	Labetolol
	Esmolol
Our case	Nicardipine
	Esmolol

The management of undiagnosed pheochromocytoma it's a veritable challenge for anaesthetist. Any patient with grossly exaggerated and unexplained hypertension and tachycardia during anaesthesia needs to be followed up and investigated for pheochromocytoma. Drugs must be available in all the anaesthetic sites and all the anaesthetists must be familiar of their uses.

## Competing interests

The authors declare that they have no competing interests.
